# Evidence of the unidimensional structure of mind perception

**DOI:** 10.1038/s41598-022-23047-6

**Published:** 2022-11-08

**Authors:** Kallie Tzelios, Lisa A. Williams, John Omerod, Eliza Bliss-Moreau

**Affiliations:** 1 School of Psychology, UNSW Sydney, Sydney, Australia; 2 School of Mathematics and Statistics, University of Sydney, Sydney, Australia; 3 Department of Psychology, California National Primate Research Center, University of California Davis, Davis, USA

**Keywords:** Psychology, Human behaviour

## Abstract

The last decade has witnessed intense interest in how people perceive the minds of other entities (humans, non-human animals, and non-living objects and forces) and how this perception impacts behavior. Despite the attention paid to the topic, the psychological structure of mind perception—that is, the underlying properties that account for variance across judgements of entities—is not clear and extant reports conflict in terms of how to understand the structure. In the present research, we evaluated the psychological structure of mind perception by having participants evaluate a wide array of human, non-human animal, and non-animal entities. Using an entirely within-participants design, varied measurement approaches, and data-driven analyses, four studies demonstrated that mind perception is best conceptualized along a single dimension.

## Introduction

One of the hallmarks of human cognition is the ability to extract information about others’ goals, beliefs, feelings, and thoughts upon simply looking at, listening to, or interacting with them; that is, mind perception. Accumulating evidence demonstrates that humans automatically and effortlessly perceive the minds of other people and nonhuman entities^1,2^, but the inherent psychological structure of mind perception is not firmly established. We use the term ‘mind perception’ to capture both the granting and denial of a range of capacities to human and nonhuman entities. As such, this is inclusive of anthropomorphism (the granting of humanlike qualities to nonhuman entities^3,4^), dehumanization (the denial of humanlike capacities to human entities^5^), and emotion ascription (the granting of emotion experience capability to an entity^6–9^). The fundamental properties used to make mind perception judgements has important real-world implications for decision-making processes across a multitude of domains including health and lifestyle decisions^10,11^, technology^12,13^, and social attitudes^14–17^.

Inferences about another entity’s mind are multifaceted—including features of bodily states, cognition, and emotion, the extent to which the entity is influenced by or influences the environment, the capacity for the entity to care for others or require care itself, and more. These capacities are interrelated such that they can be organized along one or more broad dimension(s) that represent the underlying organizing principles of the specific capacities. Published reports variably discuss mind perception as being described by one, two, or three dimensions. For example, several lines of research conceptualize mind perception as unidimensional^18–26^, spanning low to high capacity for mind. Inanimate entities (e.g., dead person) typically occupy the lower end, and adult humans the higher end^18,25^.

A large number of studies identify two dimensions of mind perception, typically comprising one that represents an entity’s capacity to have agency (e.g., think, plan, and act) and another that represents an entity’s capacity to experience the world (e.g., emote, sense, and perceive^10,27–37^). Still other studies suggest that mind perception is best characterized by three dimensions, though the nature of those dimensions varies across studies (e.g., emotion, intention, and cognition^38–41^; rationality, social impact, and valence^42^; body, heart, and mind^43–45^).

At first blush, identifying the psychological structure of mind perception may seem like a purely academic endeavor. However, how people perceive the minds of others has substantive real-world consequences. For example, attributing mind impacts decision-making and judgments in the context of: withdrawal of life support from terminally ill patients^10^, decisions to eat meat^11^, ascription of responsibility to autonomous machines^12^, endorsement of aid^15^, customer satisfaction with service robots^13^, willingness to help organizations when they suffer^14^, and perceptions of immortality after death^16^. Relatedly, denial of mind underpins support for genocide^46,47^ and mediates the effect of personal moral values on prejudice towards sexual outgroups^17^. Decisions large and small are governed by how we perceive the minds of others. The development of interventions to improve human and nonhuman animal lives rests on astute understanding of how we perceive minds. Thus, understanding the inherent psychological organization of this process is essential if society is to develop effective policies and practice that optimize wellbeing of individuals and groups alike.

It is perhaps the importance of mind perception for social processes writ large that inspires so many studies on the psychological structure of the process. The existing literature is hugely varied in terms of methods and analytic approaches, as well as the results garnered and the psychological structures they suggest. Here, we highlight three sources of variation: what and how many entities are evaluated in a given study, how evaluations are made, and how resulting data are analyzed.

With very few exceptions, studies typically include a small number of entities. For example, H. Gray and colleagues’^27^ foundational study used 13 entities: eight human entities, three nonhuman animal entities, and two non-animal entities (see also^43^). Other studies used fewer still: ten (one human entity, four nonhuman animal entities, and five non-animal entities^45^, nine (four human entities, one nonhuman animal entity, and four non-animal entities^18,48^), seven (two human entities, one nonhuman animal entity, and four non-animal entities^41^), three (“animals,” “robots (machines),” and “supernatural beings”^28^), or two (adult man, adult woman^43,49^). Research focusing on a particular phenomenon sometimes utilizes a single entity (e.g., human–robot interactions^33,50^), whereas other research includes a larger number of entities (e.g., *n* = 32^11^^Study1^, *n* = 21^43^^Study4^, *n* = 36^51^). While it is not entirely clear what the impact of such variability might be on the derived structure of mind perception, it is certainly the case that under- or over-sampling of a particular type of entity could reduce the generalizability of the derived solution.

Another methodological feature of prior work that could impact the derived structure of mind perception is how participants are asked to ascribe mind-related capacities to entities. Some studies deploy between-participant designs wherein a given participant rates only one entity on several capacities^43^^Study 1, 2, and 4,^^37^^Studies 1 and 2,^^36,45,51^ or several entities on one capacity^27^ and data are aggregated across participants^27,36,37,43,51^. Other studies provide insufficient detail to determine whether participants are carrying out the evaluations by-entity or by-capacity^11,31^, while others include questions that vary in both capacity and entity^26^. Another methodological design feature asks participants to rate entities in pairs concurrently^27,43^^Study 3,^^49^, leaving open the possibly that comparative processes could shape evaluations. Given contextual effects that one might expect if presented with multiple entities or capacities when making a judgment, variation in methodology might generate variation in the derived psychological structure of mind perception.

Finally, studies also vary in their statistical approach. Exploratory approaches allow for any number of dimensions to emerge. Indeed, past work deploying exploratory approaches yields variable numbers of mind perception dimensions^27,41,43–45,51^. Of studies that explicitly test dimensional structures, most do not specify whether the approaches were confirmatory or exploratory^11,31,33,34,38,40,41,49^^.^^cf.26,48,52^. Confirmatory approaches of course are driven by a priori assumptions about the psychological structure of mind perception thus constraining derived solutions.

In the current report, we detail a set of four studies that represent a systematic approach to identifying the psychological structure of mind perception—resolving and/or systematically varying the methodological and analytical points raised above. First, we tested a fairly large set of entities that included approximately equal numbers of human (Studies 1, 2a, and 2b: *n* = 14, Study 3: *n* = 17), nonhuman animal (*n* = 12), and non-animal (*n* = 14) entities. We sampled a range of developmental phases for humans (e.g., infant, teenager, adult, elderly) and a variety of species and environmental contexts for nonhuman animals. We also sampled inanimate objects, organizational institutions, living non-animals, and natural and supernatural forces to create a diverse sample of non-animal entities. Visual depictions of some human entities were gendered (i.e., male: child, adult; female: teenager, elderly), whereas the younger human entities (e.g., fetus, infant), the human entities with identity characteristics (e.g., blind person, identity thief), and the nonhuman animal and non-animal entities were not gendered. The full list of entities used in the present research appears in Table 1.

**Table 1:** Entities used across Studies 1, 2a, 2b, and 3.

Human	Nonhuman Animal	Non-animal
6 month old fetus	Cat	Bacteria
Blind person	Chimpanzee	Chair
Braindead person	Cockroach	Court of law
Dead person	Dog	Cyclone/hurricane
Elderly	Dolphin	God/a higher power
Fertilized human egg	Elephant	Google
Human adult	Fish	International Red Cross
Human child	Gorilla	iPhone’s Siri
Human infant	Mouse	Nature
Identity thief^a^	Rabbit	Rock
Immigrant^a^	Sparrow	The United Nations (UN)
Murderer	Whale	Tree
Person with dementia		University
Person with drug addiction		Virus
Robber/burglar^a^		
Teenager		
You		

Entities marked with ^a^ were used in Study 3 only. Terms here reflect the verbal labels that accompanied each visual depiction.

Second, we varied the formatting of questions by either asking participants to rate each individual entity on the entire set of capacities before rating another entity (i.e., a *by-entity* approach; Studies 1 and 2a) or to rate all entities on a given capacity before rating all entities on another capacity (i.e., a *by-capacity* approach; Studies 2b and 3). In all studies, this yielded a fully within-participants design in which all participants rated all entities on all capacities.

Finally, we adopted a data-driven analytic approach to explore the nature of mind perception. We deployed nine different dimensional analysis methods on non-parametric bootstrapped samples of each of the four datasets to determine consensus on the structure of mind perception. This approach offers an advantage over traditional static factor analytic techniques by maximizing the robustness of conclusions both in terms of utilization of the raw data and in terms of model assumptions. Mirroring previous research, we also deployed static exploratory principal components analyses (PCA) on all four data sets, fully reported in Supplementary Materials.

Data, R scripts, and survey programming materials are available at https://osf.io/e7wuh/. No data were excluded. Individual difference measures were not relevant to the research question and analyses reported herein, thus were not analyzed. Sample sizes were governed by time constraints of data collection. Our fully within-participants design resulted in 9120–40,248 evaluations per study. All study procedures were approved by the UNSW Sydney Human Research Ethics Approval Panel C (File #2182). All research was performed in accordance with relevant guidelines/regulations, and informed consent was obtained from all participants and/or their legal guardians.

## Results

Table 2 presents the number of principal component (PC) dimensions identified by the bootstrapping process for each of the nine dimensional analysis methods for Studies 1, 2a, 2b, and 3. Eight of the nine methods identified one PC dimension in the data from Study 1 (i.e., for eight of the methods, the majority of the 500 bootstrapped iterations pointed to one dimension). The remaining method (*t test*) identified two dimensions. As such, the consensus of these various extraction methods was a single dimension. Similar results held for Study 2a. All nine extraction methods identified one PC dimension in the data from Studies 2b and 3. This is consistent with the results of traditional, static exploratory principal components analyses, in which one component explained 84.78% (Study 1), 83.29% (Study 2a), 82.20% (Study 2b), and 79.69% (Study 3) of the variance. See Supplementary Materials for a full report of these results. In sum, across studies, methodological variations (e.g., capacities, entities, measurement approach), and analytic approaches, the results strongly point to a single dimension as the best solution capturing mind perception.

**Table 2:** Number of principal component dimensions identified by the nine dimensional analysis methods for Studies 1, 2a, 2b, and 3.

Number of dimensions identified	Dimensional analysis method								
	rndLambda	Broken stick	Twice mean	Spectral	Kmeans1	Kmeans3	T-test	T-test2	CPT
**Study 1**									
1 dimension	**500**	**500**	**500**	**500**	**500**	**500**	1	**500**	**500**
2 dimensions	0	0	0	0	0	0	**326**	0	0
3 dimensions	0	0	0	0	0	0	147	0	0
4 dimensions	0	0	0	0	0	0	22	0	0
5 dimensions	0	0	0	0	0	0	2	0	0
6 dimensions	0	0	0	0	0	0	2	0	0
**Study 2a**									
1 dimension	**500**	**500**	**500**	**500**	**500**	**500**	191	**500**	**500**
2 dimensions	0	0	0	0	0	0	**216**	0	0
3 dimensions	0	0	0	0	0	0	93	0	0
**Study 2b**									
1 dimension	**500**	**500**	**500**	**342**	**491**	**491**	**498**	**500**	**491**
2 dimensions	0	0	0	158	9	9	2	0	9
**Study 3**									
1 dimension	**500**	**500**	**500**	**500**	**500**	**500**	**464**	**500**	**500**
2 dimensions	0	0	0	0	0	0	36	0	0

Each column presents the count of the 500 bootstrapped iterations for a given method that indicated a given number of dimensions. Bold numbers represent the majority of the bootstrapped iterations.

**Figure 1:**
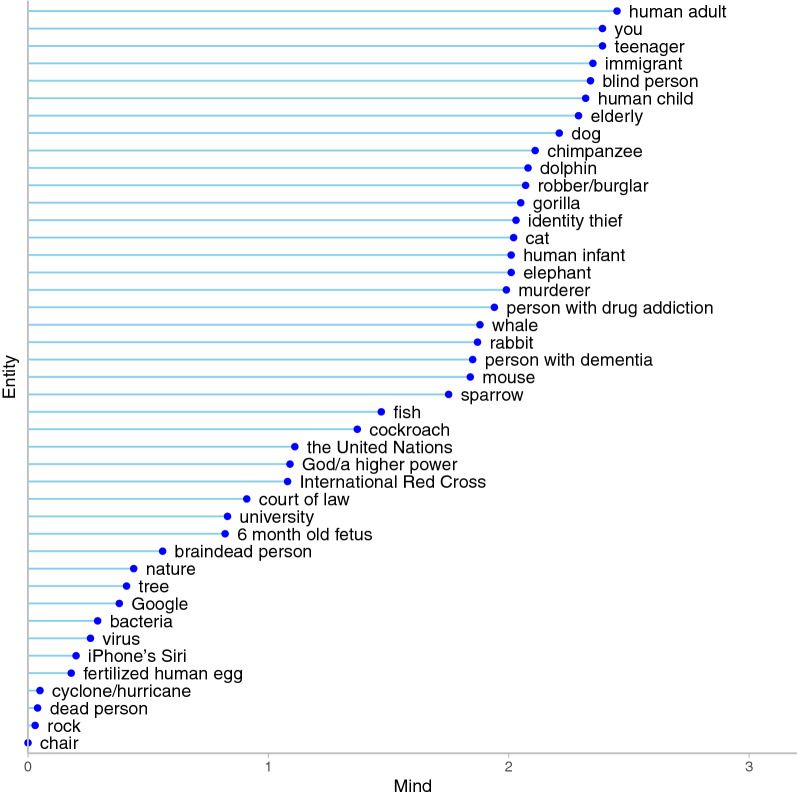
Distribution of entities along the single dimension of mind. The lines represent the average regression weights for entities from Study 3, scaled such that the lowest value corresponds to 0. A longer line represents more ascription of mind (e.g., “adult”) whereas a shorter line represents less ascription of mind (e.g., “rock”).

Average regression weights on the single dimension for each entity by study are presented in Table S2. Figure 1 depicts the average regression weight for each entity for Study 3. To facilitate interpretation of relative placement, values were transformed such that the lowest regression weight corresponded to 0. As seen, entities such as “rock” are at the low end on the single mind dimension and entities such as “adult human” are at the upper end of the mind dimension.

## Discussion

Across four studies, we demonstrated that the psychological structure of mind perception organizes into one dimension, the anchors of which can be interpreted as ranging from ‘no mind’ (e.g., chair, dead person, rock) to ‘highly developed mind’ (e.g., adult human, ‘you’). A wide variety of entities distributed along this dimension, including humans across a range of developmental stages, a host of animals that cover a broad swath of phylogeny, and entities related to technology, society, and forces of nature. The consistent and robust emergence of a unidimensional structure of mind perception when adopting an exploratory dimensional analysis approach strongly suggests that a single dimension best captures the way that individuals conceptualize mind in other entities, echoing prior work^18–21,23–25^.

The motivation to carry out this research was, in part, to resolve unanswered questions about the impact of varying mixed methodologies and analytic approaches in the extant literature. Results across the present studies were strikingly consistent: changes to the measurement approach (by-entity vs. by-capacity) and to the included entities (starting from an already expanded set of entities and adding more in Study 3) failed to produce substantive differences in the derived solutions. Moreover, consistency emerged regardless of slight changes in the number and content of measured capacities in the present studies (e.g., from nine to six; from those that include/exclude emotional states). Weisman and colleagues^43^ offer a complementary discussion of the impact of design on derived structures of mind perception.

The use of nine dimensional analysis methods applied to non-parametric bootstrapped data allowed for deployment of a consensus approach to identifying the number of dimensions that characterize mind perception. This approach allowed data to speak for themselves—which they did loudly: mind perception is best captured by a singular component/dimension. The present results offer a further cautionary tale: if researchers apply a priori assumptions about the structure of mind perception rather than adopting a data-driven approach, they may confirm a multi-dimensional structure, even in cases in which the most appropriate structure is unidimensional. Moreover, researchers should examine the structure empirically; skipping such a step may very well lead to conclusions that fail to capture the true psychological nature of mind perception.

Of course, the present studies are not without limitations. Namely, in expanding the entity set, we were constrained to using a small set of capacities (i.e., 11 capacities across the four studies) in order to maintain a reasonable task length for participants. Due to our fully within-participants design in which all participants rated all entities on all capacities in a single laboratory session, practical considerations of time and attention span limited the number of capacities that could be included (i.e., even with six capacities, participants completed 240 ratings each). Ultimately, the capacities we selected and used in this work may not comprehensively capture the breadth and complexity of mental life.

Indeed, inclusion of a greater number of capacities might shift the observed structure. For example, since we collected our data, Weisman and colleagues^43^ conducted a noteworthy line of research using 40 capacities pertaining to affective functions (e.g., calm, angry), agency (e.g., free-will, self-restraint), perceptual functions (e.g., seeing, smelling), cognitive abilities (e.g., remembering, reasoning), and physiological functions (e.g., hunger, pain). Across several studies utilizing two to 21 entities, three dimensions emerged: body states, emotions, and cognitions. While internally consistent, this solution fails to align with other three-dimensional solutions in the literature^38,40,41,44^, and of course with the popular, two-dimensional conceptualization of mind perception comprising ‘agency’ and ‘experience’^1,27^. More recent research obtaining ratings regarding 23 capacities with culturally-diverse samples reveals that this three-dimension solution holds in samples from the United States, but not as well among samples from other countries^45^. Further, Callahan and colleagues^51^ obtained ratings regarding 40 capacities in relation to 36 nonhuman animals. Exploratory factor analyses revealed two factors, comprising emotive traits (e.g., guilt, shame, and imagination) and cognitive traits (e.g., helping members of their own species, intelligence, and solving a problem with multiple stems). In light of these recent findings, comprehensive approaches are needed. For instance, future studies might incorporate the capacities from Weisman and colleagues^43,45^ and Callahan and colleagues^51^ with the more inclusive set of human, nonhuman animal, and nonanimal entities deployed in the present research and/or systematically manipulate inclusion vs. exclusion of capacities and entities to determine effects on derived structure. Such work might require multi-session approaches to maintain the advantages of fully within-participants designs and not tax participants’ attention within a single session.

Another limitation of our work is that our sample demographics are limited. While use of samples from predominantly White, Educated, Industrialized, Rich, Democratic (WEIRD) locations—as done in the present studies—is common in this area of research^10,20,23,24,26–29,37–39,48,49^ and in psychology and the behavioral sciences more broadly^53^, it is imperative that future research recruit more inclusive samples and explore the potential that demographic characteristics might moderate the inherent structure of mind^45^ (as they do more general mind perception processes^54–56^). It is also important to recognize that approaching the question of the structure and inherent qualities of mind from disciplines other than psychology is fruitful. For instance, philosophical traditions offer insights into the nature of human and nonhuman mind^57–59^ that complement psychological approaches. Multidisciplinary work across psychology, philosophy, and other disciplines carries promise for advancing understanding of the nature of mind.

In terms of our statistical analysis, we relied on principal component analysis and factor analysis methods to determine an appropriate dimension for our data. These methods may be viewed as linear dimension reduction techniques, and do not pursue nonlinear mappings of the data to low dimensional spaces. Further these methods are statistical models, not mechanistic models of the underlying phenomena and merely seek an appropriate dimension for the data at hand. Deployment of different statistical approaches to different types of data may well yield different solutions.

Mind perception impacts real-world outcomes, making the present work important—especially in the context of that which has come before it. From first impressions to human–machine interfaces, and from human-animal interactions to intractable social conflicts, the psychological processes of mind perception permeate everyday life. Whether those processes are singular or multidimensional in structure has substantive implications for the design of our future world, be it with regard to interactions with other humans, nonhuman animals, and/or technology. The present research highlights the importance of pinpointing that structure before translating findings into policy or practice.

## Method

### Participants

Undergraduate psychology students participated in all studies in exchange for partial course credit (Study 1 N = 37, Studies 2a and 2b *N* = 77, Study 3 N = 156). Studies were run independently, barring Studies 2a and 2b, in which random assignment after recruitment determined which sub-study participants completed (Study 2a *n* = 39, Study 2b *n* = 38). Sample demographics by study are reported in Table S1. Generally, the samples were predominantly female, White/Caucasian or North East Asian, and 19–20 years old.

### Procedure

Participants completed the survey, programmed in Qualtrics, in the laboratory. Full survey documents are available on the project OSF site: https://osf.io/e7wuh/. After providing informed consent, participants were told that they would be rating a number of entities, each represented with a verbal label and a line drawing. Participants were asked to consider the broad category represented by the verbal label and to treat the line drawing as an example of the category. The entities spanned human, nonhuman animal, and non-animal categories. Studies 1, 2a, and 2b utilized 40 entities. Study 3 included an additional three human entities which represented potentially marginalized types of people who may be targets of dehumanization (i.e., people who have immigrated and people who have committed robbery and identity theft^60,61^). See Table 1 for a full list of entities.

Participants rated all entities on the degree to which they were perceived to be capable of possessing a number of capacities. Capacities were selected and adapted from previous literature, with differences across studies adopted in order systematically evaluate if heterogeneity of included capacities influenced derived solutions. As noted, emotion ascription (i.e., granting an entity the capacity to experience emotion) is but one part of mind perception. Systematically including vs. excluding emotion ascription across studies allowed us to identify if doing so influenced derived solutions (e.g., does inclusion of emotion ascription lead to a solution with more [or perhaps fewer] dimensions?). As such, Studies 1, 2a, and 2b did not include capacities tapping into emotion ascription, whereas Study 3 did.

Specifically, four of six capacities utilized in K. Gray, Jenkins, Heberlein, and Wegner^48^ were used in all four studies: remembering (*memory*), exercising self-control (*self-control*), acting morally (*morality*), and feeling hunger (*hunger*). In Study 3, participants also rated entities’ capacity to feel fear (*fear*) and feel pleasure (*pleasure*), also drawn from Gray et al.^48^ and capturing emotion ascription*.* We replaced these with feeling pain (*pain*) and feeling desire (*desire*) in Studies 1, 2a, and 2b. Study 1 also included an additional three capacities with the aim of capturing a broader array of mental capacities: sensing its environment (*sense*), being aware of the passage of time (*time*), and perceiving its surroundings (*perceive*). Thus, across studies, we included a set of capacities used in prior research (Study 3), a modified set of capacities (Studies 2a and 2b), and an expanded, modified set of capacities (Study 1).

Participants rated how capable each entity was of possessing each capacity using a 7-point scale ranging from 1 (*Not at all*) to 7 (*Very Much*). In Studies 1 and 2a, participants rated each entity on all capacities prior to rating the next entity. In Studies 2b and 3, participants rated all entities on each capacity before ratings the next capacity. As such, Studies 1 and 2a used a by-entity approach, whereas Studies 2b and 3 used a by-capacity approach. The order of entities (Studies 1 and 2a) and capacities (Studies 2b and 3) was randomized across participants. After completing the entity rating task, participants provided demographic information.

### Analytic approach

Analyses were performed in R 4.0.3 programming language^62^ using the *PCDimension* package^63^. We report the results of nine principal component (PC) estimation methods. We initially used 13 methods. In light of a recent large simulation study indicating that four methods (i.e., *Bartlett’s test, Anderson, Lawley*, and *rnd-F*) perform poorly^64^, we opted to not report the results of these four methods. Here, we briefly review the nine methods and direct interested readers to Auer and Gervini^65^ for a more comprehensive review.

*rnd-Lambda* is a distribution-free method based on ter Braak^66,67^. This is a nonparametric approach where a null distribution is generated by applying the following steps a large number of times: (1) randomize the values with all the attribute columns of the data matrix; (2) perform PC analyses on the scrambled data matrix; and (3) compute the test statistics based on the PC eigenvalues. A *p*-value is determined for each dimension *k* by comparing the null distribution against the observed test statistics to determine *k*. In extensive simulation studies, Coombes and Wang^63^ and Peres-Neto, Jackson and Somers^68^ determined this method to be among the best over a variety of simulation settings.

The *broken stick* method compares eigenvalues to the expected value of the *k*th longest segment, where segments are the unit interval which is broken (uniformly) into *p* segments, *p* reflects the dimension of the data, and *k* the number of PC dimensions. The value of *k* is selected for which the *k*th largest eigenvalue exceeds the expected value of the *k*th longest segment.

The remaining methods (*twice mean*, *spectral*, *Kmeans1*, *Kmeans3*, *Ttest*, *Ttest2*, and *CPT*) are based on an Auer-Gervini model^65^. The maximum posterior estimate for the PC dimension defines a step function. The step function is then plotted and the highest dimension for which the step length is significantly large identifies the PC dimension. Each method varies in its determination of significantly large: *twice mean* is based on theory of singular value decompositions^69^, *spectral* is based on spectral clustering, *Kmeans1* and *Kmeans3* are based on the k-means clustering method, *Ttest* and *Ttest2* are based on two different *t*-test methods, and *CPT* is based on a change point method.

We used the above nine dimension estimation methods to test for the underlying PC dimension for each of the four studies. Per previous research, data were aggregated over participants before applying the PC analysis and testing for the appropriate dimension. We then bootstrapped this entire procedure using 500 bootstrap iterations in order to determine the bootstrap distribution of dimension solutions in order to determine the sensitivity of the above tests for the PC dimension. More specifically, for a particular bootstrap iteration (1) participants were drawn with replacement from a particular study until a dataset with the same number of participants was attained (a single bootstrapped dataset), (2) the data were aggregated over participants, a PCA performed, and PC testing applied to determine the appropriate dimension of the bootstrapped dataset. This procedure was repeated 500 times. Each time, the results from each PC dimension test were recorded. We sought consensus with regard to the number of dimensions that best characterized the underlying structure of the data.

Regression weights were obtained by performing a factor analysis using the *stats* package in R on the unaggregated data (without averaging the scores over each subject before performing the factor analysis). This resulted in a regression weight for each participant for each entity in each study. Entity regression weights were then aggregated over participants for each study separately.

## Supplementary Information

Supplementary Information.

## Data Availability

All data are available at: https://osf.io/e7wuh/.
