# Supplementary material for: Evidence of the unidimensional structure of mind perception
Source: Sci Rep. 2022 Nov 8;12:18978. doi: 10.1038/s41598-022-23047-6 (PMC9643359; doi:10.1038/s41598-022-23047-6)
Supplement: Supplementary file 1 — Supplementary Information. [file 41598_2022_23047_MOESM1_ESM.pdf]

Supplementary Materials for

Tzelios, Williams, Omerod, & Bliss-Moreau "Evidence of the Unidimensional Structure of Mind Perception"

Sample demographics by study

Table S1. *Sample demographics, by study*

	Study 1	Study 2a	Study 2b	Study 3
Gender				
Female	26	27	21	112
Male	11	12	17	44
Ethnicity				
North East Asian	10	9	14	57
White/Caucasian	10	16	14	39
South East Asian	9	6	5	29
South/Central Asian	2	3	4	11
Middle Eastern/North African	1	2	0	5
African/African American	1	0	0	0
"Other" or more than one	4	3	1	15
Age				
Mean	19.56	20.08	19.39	19.12
Standard deviation	3.26	2.99	2.10	2.44
Range	17-36	17-30	17-28	18-38

Regression weights per entity by study

Table S2. *Regression weights by entity for Study 1, 2a, 2b, and 3*

Entity	Study 1	Study 2a	Study 2b	Study 3
chair	-1.37	-1.26	-1.28	-1.39
rock	-1.33	-1.23	-1.28	-1.36
dead person	-1.42	-1.29	-1.24	-1.35
cyclone/hurricane	-1.25	-1.19	-1.28	-1.34
Fertilized human egg	-0.92	-1.14	-1.12	-1.21
iPhone's Siri	-0.97	-1.16	-1.09	-1.19
virus	-0.56	-1.05	-1.06	-1.13
bacteria	-0.76	-1.00	-1.03	-1.10
Google	-1.06	-1.14	-0.95	-1.01
tree	-0.75	-0.72	-0.94	-0.98
nature	-0.70	-0.68	-0.89	-0.95
braindead person	-0.87	-0.89	-0.73	-0.83
6 month old fetus	-0.34	-0.13	-0.39	-0.57
university	-0.83	-0.84	-0.46	-0.56
court of law	-0.67	-0.87	-0.37	-0.48
International Red Cross	-0.44	-0.60	-0.34	-0.31
God/a higher power	0.06	-0.50	-0.19	-0.30
The United Nations	-0.34	-0.55	-0.28	-0.28
cockroach	0.27	0.24	-0.06	-0.02
fish	0.25	0.38	0.05	0.08
sparrow	0.59	0.58	0.31	0.36
mouse	0.37	0.62	0.40	0.45
person with dementia	0.31	0.56	0.56	0.46
rabbit	0.57	0.68	0.39	0.48
whale	0.66	0.68	0.50	0.49
person with drug addiction	0.34	0.58	0.81	0.55
murderer	0.66	0.69	0.82	0.60
human infant	0.39	0.66	0.58	0.62
elephant	0.71	0.77	0.57	0.62
cat	0.70	0.76	0.57	0.63
identity thief				0.64
gorilla	0.77	0.81	0.69	0.66
robber/burglar				0.68
dolphin	0.76	0.81	0.67	0.69
chimpanzee	0.83	0.90	0.72	0.72
dog	0.82	0.88	0.69	0.82
elderly	0.83	0.87	1.02	0.90
human child	0.73	0.84	0.96	0.93
blind person	0.96	0.97	1.14	0.95
immigrant				0.96
teenager	0.91	0.97	1.12	1.00
you	0.96	1.02	1.15	1.00
human adult	1.08	0.97	1.18	1.06

Note. Entities are ordered according to Study 3 weights in ascending order.

Exploratory Factor Analysis

To mirror prior research, we carried out traditional, static factor analysis on the data from Studies 1-3. Data analysis was performed in SPSS v.25. Mean values for each question for each entity were computed across participants, resulting in a 40 (entities) x 9 (capacities) matrix. Mean ratings were subjected to principal components analysis (PCA) with varimax rotation, mirroring Gray et al. (2007). Following standard guidelines (Gorsuch, 1983), we determined the number of components based on extracted eigenvalue (cut-off = 1) and visual inspection of the scree plot. Indicators of sampling adequacy, percent variance explained, and component loadings were also evaluated and reported.

Across studies, the regression method was used to compute regression weights against derived components for each entity. Resulting weights enabled interpretation of relative placement of entities across the extracted dimensional space.

Results are reported by study below.

Study 1

The Kaiser-Meyer-Olkin Measure indicated appropriate sampling adequacy (KMO = 0.84). Individual level KMO values also indicated sampling adequacy for all capacities (all values > 0.69). Bartlett's Test of Sphericity was significant, $\chi^2(36) = 729.37$, $p < 0.001$, indicating that the correlation between capacities was sufficient for PCA. See Figure S1, Panel A for the scree plot. Table S3 presents variance explained by communalities and factor loadings. Based on the eigenvalue cutoff approach, one component that explained 84.78% of the variance was identified. Table S4 presents regression weights by entity.

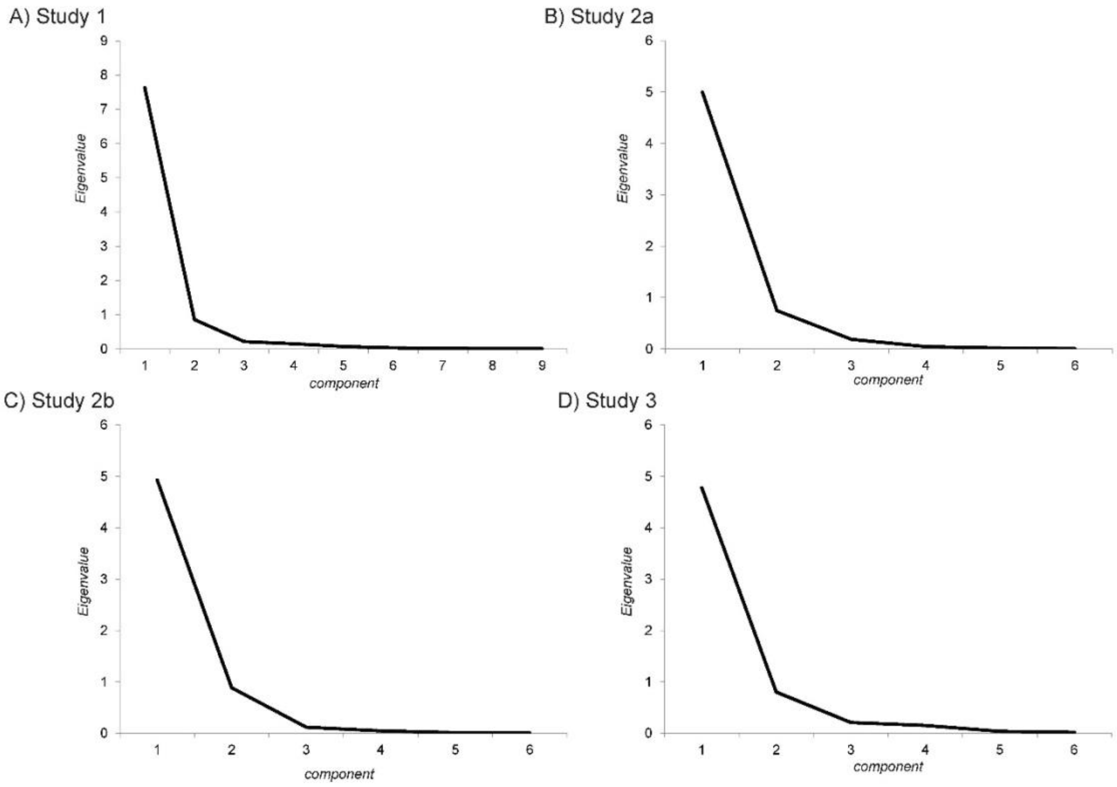

Figure 1. Scree plot for exploratory factory analysis in study 1, 2a, 2b, and 3.

Figure S1. Scree plots from Studies 1, 2a, 2b, and 3.

Table S3. *Communalities and factor loadings from Study 1*

Capacity	Communality	Factor Loading
Desire	.93	.97
Hunger	.83	.91
Memory	.90	.95
Morality	.58	.76
Pain	.89	.94
Perceive	.90	.95
Self-control	.86	.93
Sense	.88	.94
Time	.85	.92

Table S4. *Regression weights by entity for the exploratory (one-component) solution from Studies 1, 2a, 2b, and 3*

Study 1		Study 2a		Study 2b		Study 3	
Entity	Weight	Entity	Weight	Entity	Weight	Entity	Weight
chair	-1.70	cyclone/hurricane	-1.56	dead person	-1.56	chair	-1.75
dead person	-1.67	rock	-1.55	chair	-1.53	cyclone/hurricane	-1.71
rock	-1.64	chair	-1.54	rock	-1.50	dead person	-1.69
cyclone/hurricane	-1.59	dead person	-1.48	cyclone/hurricane	-1.48	fertilized human egg	-1.52
Google	-1.26	fertilized human egg	-1.40	fertilized human egg	-1.40	virus	-1.40
fertilized human egg	-1.19	virus	-1.32	virus	-1.25	bacteria	-1.36
iPhone’s Siri	-1.18	bacteria	-1.25	bacteria	-1.22	rock	-1.27
braindead person	-1.10	iPhone’s Siri	-1.19	iPhone’s Siri	-1.19	iPhone’s Siri	-1.25
bacteria	-1.02	tree	-1.17	braindead person	-1.18	tree	-1.22
university	-1.00	nature	-1.12	Google	-1.10	nature	-1.19
tree	-0.97	braindead person	-0.97	tree	-0.96	braindead person	-1.11
nature	-0.91	Google	-0.96	nature	-0.86	Google	-0.94
virus	-0.81	6 month old fetus	-0.66	university	-0.67	6 month old fetus	-0.89
court of law	-0.60	university	-0.35	6 month old fetus	-0.62	university	-0.33
6 month old fetus	-0.47	cockroach	-0.24	court of law	-0.48	cockroach	-0.26
Int’l Red Cross	-0.42	Int’l Red Cross	-0.13	Int’l Red Cross	-0.31	fish	-0.15
United Nations	-0.29	court of law	-0.10	United Nations	-0.21	court of law	-0.08
cockroach	0.16	fish	-0.08	God/a higher power	-0.17	United Nations	0.03
fish	0.21	United Nations	-0.03	cockroach	-0.03	God/a higher power	0.04
God/a higher power	0.24	God/a higher power	0.06	fish	0.12	robber/burglar	0.12
mouse	0.31	sparrow	0.27	infant	0.32	Int’l Red Cross	0.21
infant	0.36	rabbit	0.34	p.w. drug addiction	0.40	sparrow	0.26
p.w. drug addiction	0.37	mouse	0.35	mouse	0.46	mouse	0.32
p.w. dementia	0.42	infant	0.46	sparrow	0.48	infant	0.36

rabbit	0.61	whale	0.54	p.w. dementia	0.57	rabbit	0.38
sparrow	0.65	p.w. dementia	0.57	rabbit	0.60	p.w. dementia	0.39
murderer	0.76	cat	0.61	murderer	0.67	p.w. drug addiction	0.39
cat	0.77	elephant	0.65	whale	0.68	whale	0.52
whale	0.78	p.w. drug addiction	0.73	cat	0.75	murderer	0.60
elephant	0.84	dog	0.79	elephant	0.84	cat	0.62
gorilla	0.91	dolphin	0.80	dolphin	0.95	identity thief	0.68
dolphin	0.92	murderer	0.82	gorilla	0.95	elephant	0.69
child	0.94	gorilla	0.82	child	0.97	gorilla	0.73
chimp	0.96	chimpanzee	0.85	dog	1.06	dolphin	0.80
dog	0.98	child	1.13	chimpanzee	1.07	chimpanzee	0.83
elderly person	1.21	elderly person	1.33	elderly person	1.26	dog	0.93
teenager	1.24	teenager	1.40	teenager	1.33	child	1.07
you	1.33	blind person	1.48	adult	1.40	elderly person	1.23
blind person	1.35	you	1.51	blind person	1.41	teenager	1.30
adult	1.50	adult	1.57	you	1.41	immigrant	1.35
						blind person	1.36
						you	1.41
						adult	1.51

Note. “p.w.” denotes “person with”.

Study 2a

The Kaiser-Meyer-Olkin Measure indicated appropriate sampling adequacy (KMO = 0.78). Individual level KMO values also indicated sampling adequacy for all capacities (all values ≥ 0.70). Bartlett's Test of Sphericity was significant, $\chi^2(15) = 452.39$, $p < 0.001$. See Figure S1, Panel B for the scree plot. Table S5 presents variance explained by communalities and factor loadings for each capacity. The exploratory approach yielded a one-component solution that explained 83.29% of the variance. See Table S4 for regression weights by entity.

Table S5. *Communalities and factor loadings from Study 2a*

Capacity	Communality	Factor Loading
Desire	.95	.97
Hunger	.82	.91
Memory	.84	.92
Morality	.66	.81
Pain	.88	.94
Self-control	.86	.93

Study 2b

The Kaiser-Meyer-Olkin Measure indicated appropriate sampling adequacy (KMO = 0.73). Individual level KMO values also indicated sampling adequacy for all capacities (all values > 0.61). Bartlett's Test of Sphericity was significant, $\chi^2(15) = 477.22$, $p < 0.001$. See Figure S1, Panel C for the scree plot. Table S6 presents variance explained by communalities and factor loadings for each capacity. The exploratory approach yielded a one-component solution that explained 82.20% of the variance. See Table S4 for regression weights by entity.

Table S6. *Communalities and factor loadings from Study 2b*

Capacity	Communality	Factor Loading
Desire	.95	.97
Hunger	.74	.86
Memory	.88	.94
Morality	.72	.85
Pain	.82	.90
Self-control	.72	.91

The results of Studies 2a and 2b essentially mirror those of Study 1. A single component (or unidimensional solution) representing "mind" fit the data the best. Further, querying participants on a by-entity or by-capacity basis had negligible impact on the psychological structure of mind perception: the solutions across Studies 2a and 2b were remarkably similar.

Study 3

The Kaiser-Meyer-Olkin Measure indicated appropriate sampling adequacy (KMO = 0.79). Individual level KMO values also indicated sampling adequacy for all capacities (all values > 0.70). Bartlett's Test of Sphericity was significant, $\chi^2(15) = 366.87$, $p < 0.001$. See Figure S1, Panel D for the scree plot. Table S7 presents variance explained by communalities and factor loadings for each capacity. The exploratory approach yielded a one-component solution that explained 79.69% of the variance. See Table S4 for regression weights by entity. As in Studies 1, 2a, and 2b, the data from Study 3 best fit a single component/unidimensional solution representing "mind".

Table S7. *Communalities and factor loadings from Study 3*

Capacity	Communality	Factor Loading
Fear	.74	.86
Hunger	.71	.84
Memory	.82	.90
Morality	.73	.86
Pleasure	.91	.95
Self-control	.87	.93
